# Comparing right- and left sided injection-drug related infective endocarditis

**DOI:** 10.1038/s41598-020-80869-y

**Published:** 2021-01-13

**Authors:** Allan Clarelin, Magnus Rasmussen, Lars Olaison, Sigurdur Ragnarsson

**Affiliations:** 1 Division of Cardiothoracic Surgery, Department for Clinical Sciences Lund, Skane University Hospital and Lund University, Getingevagen 4, 22185 Lund, Sweden; 2 Division of Infection Medicine, Department of Clinical Sciences Lund, Medical Faculty, Lund University, Lund, Sweden; 3 Department of Infectious Diseases, Institute of Biomedicine, University of Gothenburg, Gothenburg, Sweden

**Keywords:** Bacterial infection, Valvular disease

## Abstract

The aim of the study was to compare background characteristics, microbiology and outcome of patients with right-sided and left-sided intravenous drug use (IDU) associated infective endocarditis (IE). A nationwide retrospective study using the Swedish Registry on Infective Endocarditis between 2008 and 2019 was conducted. A total of 586 people with IDU-IE were identified and divided into left-sided (n = 204) and right-sided (n = 382) IE. Descriptive statistics, Cox-regression and Kaplan–Meier survival estimates were used. The mean age of patients in the left-sided group was 46 years compared to 35 years in the right-sided group, p < 0.001. Left-sided IE had a higher proportion of females. *Staphylococcus aureus* was the causative pathogen in 48% of cases in the left-sided group compared to 88% in the right-sided group. Unadjusted and adjusted long-term survival was better in right-sided IE compared to left-sided IE. Independent predictors of long-term mortality were increasing age, end-stage renal disease, nosocomial infection, brain emboli and left-sided IE. Left-sided IE was common in people with IDU but the proportion of females with left-sided IE was low. *S. aureus* was twice as common in right-sided IE compared to left-sided IE, and the long-term prognosis of right sided IDU-associated IE was better compared to left-sided IE despite the fact that few were operated.

## Introduction

Intravenous drug use (IDU) is strongly associated with infective endocarditis (IE)^1–3^. Non-sterile injection techniques facilitate the entry of skin bacteria into the blood stream. In addition, particles other than the drugs themselves are introduced into the circulation and are thought to damage the endocardium, particularly at the right-sided heart valves^4^. People with IDU are a particularly challenging group when it comes to IE treatment due to high rates of continued drug use and recurrent IE^5,6^. The risk of death and need for valve replacement increases in cases of reinfection which further complicates the management of IE in people with IDU^7^.

Despite the fact that people with IDU-associated IE are more commonly young and do not have chronic diseases associated with early mortality, the long-term outcome of IDU-associated IE has been reported to be very poor^2^. Reinfection, drug abuse relapse, and socioeconomic challenges are thought to play a role in limiting the life span of these persons^2,8^. Although the majority of IDU-associated IE episodes affect the right side of the heart, a significant proportion of 20–30% occurs on the left side^9–11^.

There is limited information about the differences in background data and microbiological etiology between left-sided and right-sided and if there is a difference in outcome between left-sided and right-sided lesions. Chamber pressures differ between the right side and left side of the heart and calcifications occur predominantly on the left side. Indications for surgery are not the same for right-sided and left-sided IE^12,13^. Understanding differences in background characteristics and microbiology may provide a better understanding of the difference in pathophysiology of right-sided and left-sided IDU-associated IE.

The main aim of this study was to describe patient characteristics and microbiology in right-sided vs. left-sided IE in people with IDU, to identify determinants of long-term survival in IDU-associated IE patients and to compare long-term survival in patients with right-sided vs. left-sided IDU-associated IE.

## Methods

### Study design and population

**Figure 1:**
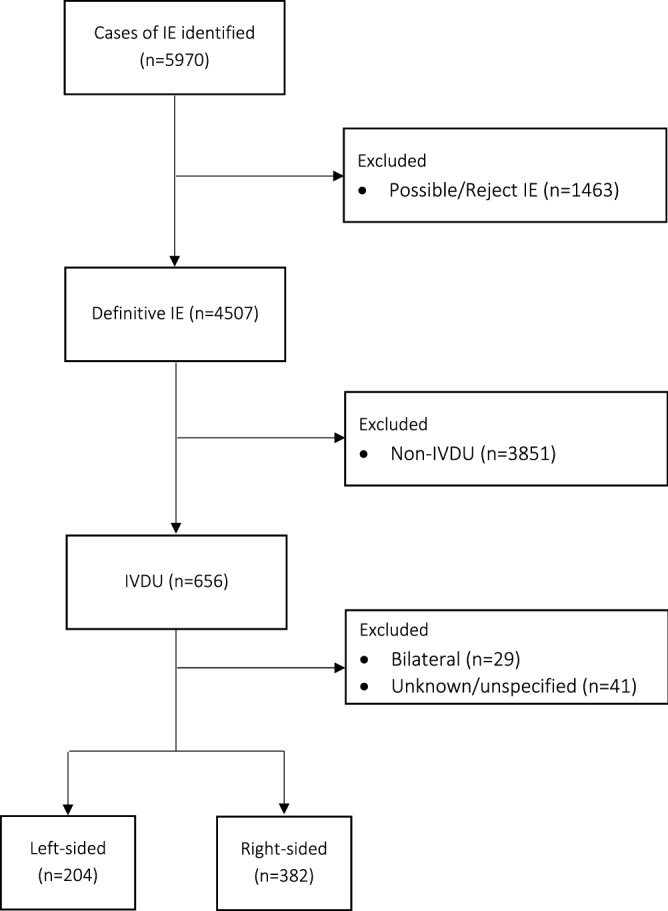
Patient selection (flow-chart). The patient selection is shown. Definitive IE was defined by Duke’s criteria. IE, infective endocarditis; IVDU, intravenous drug users.

We conducted a retrospective cohort study with data from the Swedish Registry on Infective Endocarditis (SRIE). The registry includes almost 300 variables including patient background data and comorbidities, microbiology, echocardiography, treatment data, including medical treatment and surgery. The registry variables are entered by treating physicians who are usually infectious disease specialists. The study period was from 2008 to 2019. During this period a total of 5969 episodes of IE were entered into the registry. IE was defined as definitive IE based on the modified Duke’s criteria^14^. Patients who did not have a Duke’s criteria classification in the database were classified within the present study based on the various clinical variables available in the database that constitute the Duke’s major and minor criteria. IDU-status was based on the physician’s assessment using either direct information from the patients or medical records. As seen in Fig. 1, following the exclusion of non-definitive IE and non-IDU IE a total of 656 cases of definitive IDU-IE were identified. In order to compare right-sided versus left-sided IE, we excluded patients with IE affecting both sides of the heart (n = 29), those with an unspecified or where no valve was affected (n = 41). The study population of 586 patients was divided into right-sided (n = 382) and left-sided (n = 204) IE. The primary endpoint was long-term mortality, short-term mortality was defined as ≤ 30 days. Other variables of interest were background characteristics, microbiology, IE complications including surgery and short-term mortality.

The study protocol was approved by the Swedish Ethical Review Authority (Registration number 2019-03549). As this was a retrospective study, individual consent was waivered. All methods were performed in accordance with the relevant guidelines and regulations.

### Follow-up

Follow-up on survival was done through the National Population Registry. Follow-up time was defined as the period between hospital admission and the current or latest status of the patient. Follow-up was completed on 8th of October 2019. The median follow-up time was 4.1 years (Interquartile range 1.9–6.8 years) and included a total of 2629 patient years.

### Statistical analysis

Categorical variables were presented as number and percentages. Continuous variables were normally presented as mean ± standard deviation if they were normally distributed while non-normally distributed variables were presented as median with interquartile range. Categorical variables were compared using the Chi-square test or the Fisher’s exact test if at least one of the cells had an expected count of less than five. Continuous normally distributed variables were compared with Student’s t-test while non-normally distributed variables were compared with Mann–Whitney U test. Predictors of long-term mortality were analyzed using Cox-regression. Univariable cox-regression was first calculated on variables that are known risk factors and those that were deemed important in this study. Variables that had a p-value < 0.10 were then included in the multivariate Cox-regression. Unadjusted survival was estimated using the Kaplan–Meier method Log-rank test was used to compare survival between groups. Adjusted survival was estimated with multivariable cox regression. A survival plot was performed using the same Cox-regression model as mentioned before except that IE location was a factor variable and not a covariate. The significance level was set at p < 0.05. All of the statistical analyses were completed using the statistical software program SPSS (V.26.0; IBM, Amonk, New York, USA).

## Results

### Left-sided and right-sided IE in IVDUs

Patient characteristics for left-sided IE and right-sided IE in IVDUs are listed in Table 1. Out of 586 patients, 382 had right-sided IE (65%), whereas 204 had left-sided IE (35%). The mean age in left-sided IE was 46 years, whereas in right-sided IE the mean age was 35 years (p < 0.001). Female patients were more common in right-sided IE (40% vs 23%, p < 0.001). Prosthetic valve endocarditis was more common in left-sided IE, occurring in 15% of the cases, compared to 2.1% in right-sided IE. Abscess formation occurred in 15% of the left-sided IE cases and 0.8% of the right-sided IE cases (p < 0.001). The total number of episodes with abscess that occurred in left-sided IE was 30, whereof 13 also had prosthetic valve IE. Emboli to the brain were more common in left-sided IE whereas emboli to the lungs were more common in right-sided IE. Patients with left-sided IE underwent surgery more often compared to right-sided IE (42% and 5.5%, respectively).

**Table 1:** Patient characteristics in IVDUs.

Variable	N	Left-sided IE, n = 204	Right-sided IE, n = 382	p-value
Age	585	46 (± 12)	35 (± 9)	< 0.001
Female	585	46 (23%)	152 (40%)	< 0.001
Diabetes	569	5 (2.5%)	7 (1.9%)	0.8
ESRD	570	3 (1.5%)	0 (0.0%)	0.05
Tumor disease	586	2 (1.4%)	0 (0.0%)	0.1
CIED	586	2 (1.0%)	4 (0.0%)	1
Nosocomial	586	11 (5.4%)	5 (1.3%)	0.004
Community-acquired	586	190 (93%)	367 (96%)	0.1
Prosthetic valve IE	585	30 (15%)	8 (2.1%)	< 0.001
CIED IE	586	0 (0.0%)	2 (0.5%)	0.6
Previous IE	586	70 (34%)	106 (28%)	0.1
Transthoracic echocardiogram	576	126 (64%)	268 (71%)	0.1
Transesophageal echocardiogram	574	158 (81%)	260 (69%)	0.003
Vegetation	575	187 (94%)	356 (95%)	0.5
Abscess	586	30 (15%)	3 (0.8%)	< 0.001
**Pre-existing valve disease**				
Rheumatic valve disease	586	0 (0.0%)	0 (0.0%)	
Congenital heart disease	586	1 (0.5%)	1 (0.3%)	1.0
**Emboli**				
Brain	586	39 (19%)	3 (0.8%)	< 0.001
Spondylitis	586	23 (11%)	23 (6.0%)	0.02
Lung	586	11 (5.4%)	236 (62%)	< 0.001
Other	586	53 (26%)	63 (17%)	0.006
Surgery	586	86 (42%)	21 (5.5%)	< 0.001
30-day mortality	586	14 (6.9%)	2 (0.5%)	< 0.001
In-hospital mortality	586	19 (9.3%)	2 (0.5%)	< 0.001

N represents the total number of responses in each variable.

Continuous variables (normally distributed) are presented as mean with standard deviation in parenthesis. Dichotomous variables are presented as number of cases with percentage in parenthesis.

Diabetes includes both type 1 and type 2. There were 17 missing responses for diabetes in total.

ESRD had 16 missing responses in total.

*ESRD* end-stage renal disease, *CIED* cardiovascular implantable electronic device.

### Microbiology

**Figure 2:**
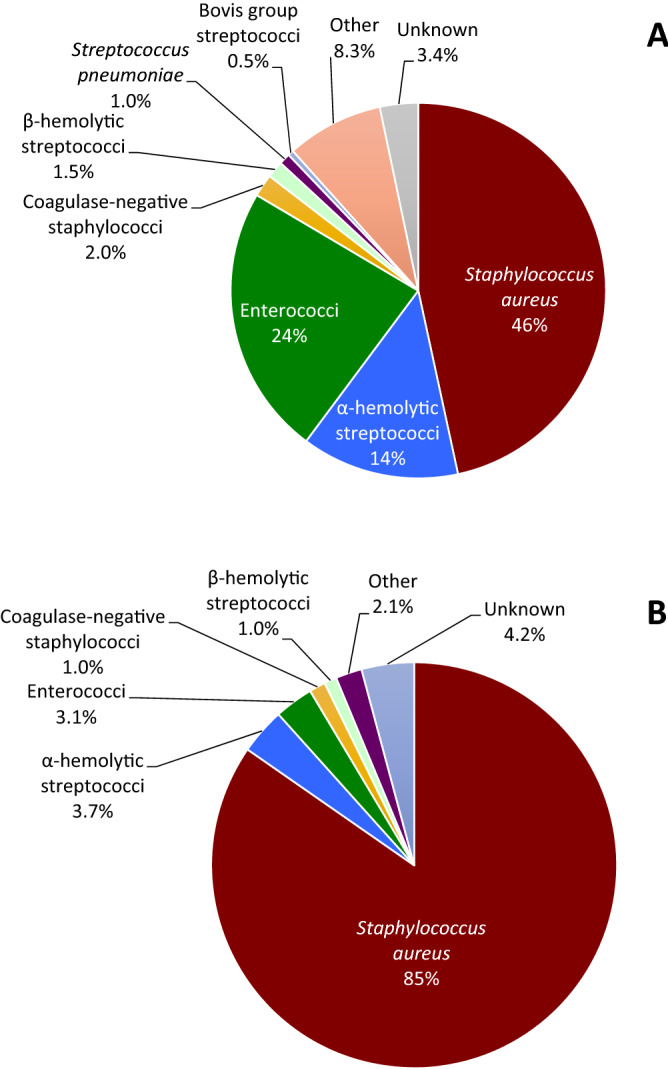
Microbiology: left-sided IDU-IE **(A)**; right-sided IDU-IE **(B)**. HACEK, *Haemophilus species, Aggregatibacter actinomycetemcomitans, Cardiobacterium hominis, Eikenella corrodens, Kingella kingae*; IDU, intravenous drug use; IE, infective endocarditis.

The causative pathogen for both left-sided and right-sided IDU-IE is summarized in Table 2 and Fig. 2. In right-sided IE, *Staphylococcus aureus* compromised 85% of the cases compared to left-sided IE where it compromised 46% of the cases (p < 0.001). Alpha-hemolytic streptococci and Enterococci were more common in left-sided IE than in right-sided IE, 14% and 3.7% vs. 24% and 3.1% respectively (p < 0.001). No difference was found for other bacteria which caused relatively few IE episodes.

**Table 2:** Microbiological aetiology in IVDUs.

Variable	Left-sided IE, n = 204	Right-sided IE, n = 382	p-value
*Staphylococcus aureus*	94 (46%)	323 (85%)	< 0.001
Alfa-hemolytic streptococci	28 (14%)	14 (3.7%)	< 0.001
Enterococci	48 (24%)	12 (3.1%)	< 0.001
Coagulase-negative staphylococci	4 (2.0%)	5 (1.3%)	0.7
Beta-hemolytic streptococci	3 (1.5%)	4 (1.0%)	0.7
*Streptococcus pneumoniae*	2 (1.0%)	0 (0%)	0.1
Bovis group streptococci	1 (0.5%)	0 (0%)	0.4
HACEK	0 (0%)	0 (0%)	
Other pathogens	17 (8.3%)	8 (2.1%)	< 0.001
Pathogen unknown^a^	7 (3.4%)	16 (4.2%)	0.7

*S. aureus* includes both methicillin-sensitive (n = 411, 98.5%) and methicillin-resistant (n = 6, 1.5%).

Alpha-hemolytic streptococci includes digestive, oral and unclassified alpha-hemolytic streptococci.

Enterococci includes *Enterococcus faecium*, *Enterococcus faecalis* and unclassified enterococcal species.

Beta-hemolytic streptococci includes group A, B, C, F, and G.

Other pathogens include other gram-positive and gram-negative bacteria and fungi.

HACEK, *Haemophilus* species*, Aggregatibacter actinomycetemcomitans, Cardiobacterium hominis, Eikenella corrodens, Kingella kingae*; *IE* infective endocarditis.

^a^Pathogen unknown include patients without positive blood cultures and those where a pathogen was not identified with other means such as tissue culture or PCR of a valve specimen.

### Long-term survival and predictors of long-term mortality

**Figure 3:**
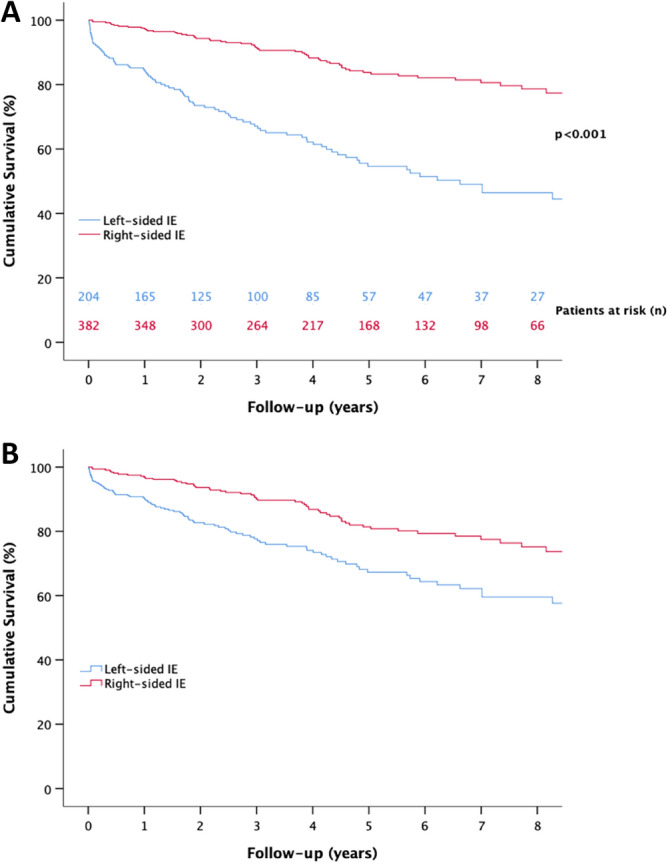
**(a)** Kaplan Meier estimate of long-term survival**, (b)** Cox-regression adjusted long-term survival. **(a)** Survival in people with IDU, for left-sided and right-sided IE. *IE* infective endocarditis. Kaplan–Meier curves are shown. (**b)** Survival in people with IDU, for left-sided and right-sided IE when adjusted for covariates. *IE* infective endocarditis. Cox-regression plot curves are shown**.**

The unadjusted survival rates after 1-year and 5-year follow-up were 85% (95% confidence interval [CI] 80% to 90%) and 55% (95% CI 47% to 63%) for left-sided IE, respectively. The survival rates for right-sided IE in the same follow-up time were 97% (95% CI 95% to 99%) and 84% (95% CI 80% to 88%), respectively. The long-term survival was thus significantly worse in left-sided IE compared to right-sided IE (Fig. 3a, log rank p < 0.001). Predictors of long-term mortality in people with IDU-IE are presented in Table 3. Age, end-stage renal disease, nosocomial infection, emboli to the brain and left-sided IE were independent predictors of long-term mortality in IDU-IE. The estimated survival adjusted for age, end-stage renal disease, nosocomial infection, and brain emboli is shown in Fig. 3b.

**Table 3:** Univariable and multivariable Cox regression for predictors of long-term survival in patients with left-sided and right-sided IVDU associated IE.

Predictor	Univariable analysis			Multivariable analysis		
	Wald	p-value	OR (95% CI)	Wald	p-value	OR (95% CI)
Age^a^ (per year)	46.5	< 0.001	1.05 (1.04–1.07)	11.3	0.001	1.03 (1.01–1.05)
Female sex	3.3	0.07	0.72 (0.50–1.03)	0.06	0.8	1.05 (0.71–1.56)
Diabetes	0.04	0.8	1.1 (0.4–3.0)			
ESRD	15.2	< 0.001	9.9 (3.1–31.5)	4.7	0.03	4.4 (1.2–16.4)
Tumor disease	5.3	0.02	5.2 (1.3–21.0)	0.17	0.7	1.37 (0.30–6.32)
ICD/Pacemaker	3.4	0.07	2.9 (0.9–9.3)	2.3	0.1	2.71 (0.75–9.79)
Nosocomial	14.9	< 0.001	3.6 (1.9–6.8)	6.4	0.01	2.4 (1.2–4.9)
Prosthetic valve IE	32.4	< 0.001	4.0 (2.5–6.5)	3.6	0.06	1.82 (0.98–3.37)
Previous IE	6.9	0.009	1.6 (1.1–2.2)	1.2	0.3	1.24 (0.85–1.81)
Vegetation	0.007	0.9	0.97 (0.50–1.91)			
Abscess	13.8	< 0.001	2.8 (1.6–4.7)	0.01	0.9	1.04 (0.54–2.01)
Brain emboli	29.6	< 0.001	3.3 (2.2–5.1)	3.9	0.05	1.62 (1.00–2.63)
Pathogen	16.6	0.001		0.34	1	
Alpha-hemolytic streptococci (ref)^b^	Ref	Ref	Ref	Ref	Ref	Ref
*S. aureus*	4.8	0.03	0.53 (0.30–0.94)	0.001	1	0.99 (0.52–1.87)
Enterococci	0.08	0.8	1.1 (0.6–2.1)	0.19	0.7	0.86 (0.42–1.74)
Other	0.01	0.9	1.0 (0.5–2.0)	0.01	0.9	0.96 (0.48–1.95)
Mitral involvement	24.0	< 0.001	2.3 (1.7–3.3)	0.20	0.7	0.90 (0.56–1.45)
Surgery	18.2	< 0.001	2.1 (1.5–3.1)	1.0	0.3	1.26 (0.81–1.95)
Left-sided IE	58.6	< 0.001	3.6 (2.6–5.1)	6.0	0.01	2.1 (1.2–3.7)

Diabetes includes both type 1 and type 2 diabetes mellitus.

No patients had a background of ESRD or tumor disease in right-sided IE.

Alpha-hemolytic streptococci were used as reference for pathogens.

*ESRD* end-stage renal disease, *ICD* intra cardiac device, *IE* infective endocarditis.

^a^Increasing age per 1-year increment.

^b^Alpha-hemolytic streptococci was used as reference in the analysis.

## Discussion

The main findings in this study are that more than one-third of people with IDU had left-sided IE, the proportion of female patients with left-sided IE was much lower than the proportion of female patients with right-sided IE, and that the long-term prognosis of right sided IDU-associated IE was better compared to left-sided IE despite the fact that only 5.5% were operated.

The current understanding of the pathophysiology of IE is that both bacteremia and endothelial damage are required for bacteria to attach to endothelium and multiply^15^. In right-sided IDU-associated IE, both requirements are met when injected particles damage the right-sided valves and when skin bacteria are introduced in the blood stream^16^. Particles injected into the blood are less likely to damage left-sided valves as they are more likely to get trapped in the lungs. Thus, left-sided IE is more likely to develop in the presence of underlying valve diseases that facilitate endothelial damage and the prevalence of degenerative valve disease increases with advanced age^17^. Epidemiological studies show that people with IDU in high-income countries are significantly older than those in low-income countries^18^. Furthermore, the mean age of people with IDU has been increasing in recent years^19^, which may further shift the trend towards left-sided IDU-associated IE. Our result show that more than a third of all IDU-associated IE reported to SRIE had a left-sided infection. The high frequency of left-sided involvement has previously been documented by Mathew et al., where the different sites had approximately equal frequency^20^. The mean age of the group with left-sided IE was 11 years higher than that of the right-sided group, which may reflect a higher burden of degenerative valve disease in the left-sided group. The presence of prosthetic valves is also an important substrate for IE. The significantly higher proportion of prosthetic valve IE in left-sided IE likely reflects that prosthetic valves are more common in the left side of the heart.

Our study showed that less than a quarter of left-sided IDU-associated IE were female while the proportion of right-sided patients who were female was 40%. The overall proportion of female patients with IDU-associated IE has been reported to be between 44 and 51%^21,22^. Previous studies on IDU-associated IE have not stratified patients according to left or right sided location. The reason for the difference in sex distribution in our cohort is unknown. However, the notion that left-sided IDU-associated IE is more similar to non IDU-associated IE than right-sided IDU-associated IE could hold true for sex distribution. There is a clear male predominance when it comes to valve surgery for degenerative aortic valve disease^23^ and degenerative mitral valve disease^17,24^. Many of the valve lesions that are most commonly operated, are also potential substrates for endothelial damage that in conjunction with IDU-associated bacteremia may cause IE. In addition to this, male patients are more likely to have cardiovascular disease at a younger age compared to their female counterparts^25^. The protective effects estrogen active until menopause could further explain the male predominance in valve surgery.

Surgery was almost eight times more likely in left-sided IE compared to right-sided IE. We believe that the reason for this is multifactorial. First, the indications for surgery are different in right-sided IE and left-sided IE^12,13^. Invasiveness and abscess formation, which constitute surgical indication^12^, primarily occur on the left side of the heart. Furthermore, tricuspid valve insufficiency is better tolerated than both aortic valve and mitral valve insufficiencies. Indeed, isolated tricuspid valve valvectomy has been reported to be used as a treatment modality in patients that are not deemed suitable for tricuspid valve replacement with favorable results^26^.

*S. aureus* was the predominant causative agent in right-sided IE and other pathogens accounted for a minority of episodes. *S. aureus* is very common in cutaneous portal of entry and is more likely to occur with poor injection hygiene. It is therefore not surprising that vast majority of right-sided IDU-associated IE are caused by this bacterium and that it is also the most common bacterium in IDU-associated IE on the left side. In left-sided IE the causative pathogens resembled more what could be expected in non-IDU-associated IE in left-sided IE, where a large proportion of the cases are caused by alpha streptococci and enterococci^27^. One explanation may be the different virulence factors expressed by the bacteria where some pathogens prefer certain conditions that may differ between the heart valves depending on their virulence factors. Pressure, flow, structure of the valves as well as calcification status differs between the left side and right side of the heart. Calcium deposits in the aortic valve and mitral annulus increase with age but are usually not seen on the right side of the heart. Thus, it does not surprise that these factors may predispose for certain microorganisms such as *S. aureus* and enterococci to attach to the surface of different heart valves^28,29^.

Left-sided IE was associated with worse long-term outcome than right-sided IE and at five years, 45% had died in the group with left-sided IE compared to 15% in the right-sided group. This was despite the fact that patients with left-sided IE were operated much more frequently than patients with right-sided IE (42% vs 5.5%). One explanation may be that left-sided infections more often is complicated by severe heart failure, systemic embolization and uncontrolled infection whereas invasiveness is uncommon in right-sided involvement^30^ and lesions in the low-pressure right side are better tolerated than lesions on the left side^31^. Indeed, in multivariable cox regression, left-sided infection was an independent predictor of long-term mortality, suggesting that it is more favorable for people with IDU to suffer a right-sided infection. Surgery was not an independent predictor of survival in this cohort. A recent meta-analysis compared IDU-associated IE with non-IDU-associated IE that underwent surgery and showed shorter long-term survival in IDU-associated IE which may signal that surgery can be problematic in people with IDU^32^. It is worth to highlight the importance of preventing return to substance use to improve survival in IDU-IE treated with surgery^33^, as this is the predominant cause of death in people with IDU-IE undergoing surgery^34^.

### Strengths and limitations

The current study includes patients reported to a nationwide registry. The patients are reported from all hospitals that have an infectious disease department, which decreases the risk of a biased patient selection that is usually seen in reports from tertiary care facilities. However, as reporting is not mandatory, the registry does not contain all IE episodes in Sweden. The retrospective nature of the study has inherent risks of biases. The IE registry does not contain more data on the nature of IDU such as what type of substances were used, and whether the patients continued IDU following surgery. There was no data regarding socioeconomic and demographic factors. People who inject drugs may also get endocarditis from non-IDU related causes such as blood stream contamination following dental surgery, this was not specified in the registry. Thus, our findings are applied to IDU-association.

## Conclusion

This study adds important information on IDU-associated IE and the differences seen in right-sided and left-sided IE in this patient group. IDU-associated IE is not limited to the right side of the heart as more than a third of the patients had left-sided IE. *S. aureus* occurred twice as much in right-sided IE compared to left-sided IE in IDU-associated IE. Survival in right-sided IE is relatively high and few patients in this group undergo surgery.
